# Editorial: Controversies in neonatal hypoglycemia

**DOI:** 10.3389/fped.2023.1236258

**Published:** 2023-06-23

**Authors:** Jeffrey R. Kaiser, Kathryn Beardsall, Deborah L. Harris

**Affiliations:** ^1^ Departments of Pediatrics (Neonatal-Perinatal Medicine) and Obstetrics and Gynecology, Penn State Children's Hospital, Milton S. Hershey Medical Center, Hershey, PA, United States; ^2^ Departments of Pediatrics, University of Cambridge, Cambridge University Hospitals NHS Foundation Trust, Cambridge, United Kingdom; ^3^ Newborn Intensive Care Unit, Waikato District Health Board, Hamilton, New Zealand; ^4^ School of Nursing, Midwifery and Health Practice, Faculty of Health, The Herenga Waka, Victoria University of Wellington, Wellington, New Zealand; ^5^ Liggins Institute, University of Auckland, Auckland, New Zealand

**Keywords:** transitional neonatal hypoglycemia, screening test, risk score, measurement bias, continuous glucose monitor (CGM), breastfeeding, hyperinsulinism

**Editorial on the Research Topic** Controversies in neonatal hypoglycemia

The conundrum of how to manage neonatal hypoglycemia continues to be plagued by multiple controversies, including its definition, whether current screening guidelines fulfill the criteria for being good screening tests, what tools should be used to measure and monitor glucose concentrations, and whether early feeding or milk composition influences glucose homeostasis. Moreover the conflation of data from a huge diversity of infants from healthy term newborns to those with variable risk factors makes interpretation of data into useful guidance for clinical practice challenging.

Robust evidence that asymptomatic transitional neonatal hypoglycemia negatively impacts neurodevelopment and whether its treatment improves outcomes is lacking. Based on these concerns, international organizations provided “eminence-based” recommendations regarding screening and management, but because of perceived too liberal or conservative treatment thresholds recommended, many institutions have developed their own guidelines. Thus, since the 1950–60s when clinical manifestations were first clearly associated with severe neonatal hypoglycemia (1), we are no further along in our understanding of the day-to-day management of hypoglycemia.

The goal of this Research Topic was to identify some of the controversies surrounding neonatal hypoglycemia that have made it difficult to develop evidence-based guidelines, or at the very least reach consensus.

The holy grail of neonatal hypoglycemia screening is the detection of neuroglycopenia, i.e., brain energy insufficiency. Alsweiler et al. concluded that current hypoglycemia screening guidelines fail to meet many of the necessary principles. Neonatal hypoglycemia is an important sign of multiple conditions, but is not a disease in itself. A recognizable latent phase, where it is possible to detect a disease before injury occurs is a necessary principle of screening, but it is unclear if this exists for the majority of hypoglycemic newborns with asymptomatic transitional hypoglycemia. The “screening test”, of a single blood glucose measurement, is not an effective proxy for neuroglycopenia. While treatment is beneficial in newborns with persistent hypoglycemia, it likely does not benefit otherwise healthy newborns with mild transitional hypoglycemia. The diagnosis of transitional hypoglycemia, however, can only be made in retrospect! Hypoglycemia screening has not been shown to reduce mortality or brain injury. Thus, further research is needed to determine which infants will benefit from updated hypoglycemia screening programs.

Another potential controversy is whether recommended glucose treatment thresholds suggested by international organizations are subject to instrument measurement bias. Duke et al. noted that the American Academy of Pediatrics (AAP) (2) and Pediatric Endocrine Society (PES) (3) based their hypoglycemia screening and management guidelines on studies that used older glucose analyzers. The authors observed that a commonly used newer glucose analyzer has an approximately negative 5 mg/dl bias compared to analyzers used to develop the AAP and PES guidelines. Thus, when managing neonatal hypoglycemia, it is critical to know which analyzer was used, and whether adjustments for potential instrument measurement bias are necessary when following published guidelines.

Risk scoring algorithms are used in clinical medicine to assess the likelihood of developing a particular outcome, such as death, need for hospitalization, or a disease, using demographic variables, signs and symptoms, and other clinically relevant factors. Ibrahim et al. developed a risk scoring algorithm to determine whether intravenous dextrose was required for resolution of hypoglycemia in hypoglycemic infants of gestational diabetic mothers. This retrospective single center study identified a hypoglycemic risk score at 1 h of age. They found that a high risk score significantly predicted the need for parenteral dextrose, and concluded that early identification of newborns who do and do not require intravenous dextrose for resolution of hypoglycemia will be helpful in triaging them to either remain with their mothers or to be transferred to units with higher levels of care.

Continuous glucose monitoring is a promising technology that may eventually supplant *intermittent* blood glucose sampling. But is its use currently “ready for prime time” in the clinical care of newborns at risk of hypoglycemia? Kalogeropoulou et al. reviewed continuous glucose monitoring use in premature and term newborns, and highlighted its potential advantages, but also noted important shortcomings that limit its use at the bedside. It was not designed nor has it been approved for use in newborns, and lacks accuracy at the lower glucose concentrations common in hypoglycemic newborns. It detects clinically silent neonatal hypoglycemia, however, in follow-up studies, silent hypoglycemia has not been linked with adverse academic performance (4). While continuous glucose monitoring provides trends over time allowing for treatment before concentrations reach dangerously low levels, the authors noted that it currently lacks the necessary accuracy to be diagnostic of specific low glucose concentrations.

The relationship between feeding and changes in glucose concentrations was evaluated by Harris et al. from GLOW Study participants (5). Contrary to recommendations by the AAP (2) regarding feeding as treatment for hypoglycemia, they observed no significant increase in interstitial glucose concentrations after breastfeeding on day 1, unless lasting >30 min. They reported increases in glucose concentrations after breastfeeding in newborns >2 days old, breastfeeding for >30 min, and feeding from both breasts. Perhaps, as suggested by the authors, recommendations about breastfeeding as treatment for hypoglycemia should consider including longer duration of breastfeeding and feeding from both breasts.

New approaches to screening and management of neonatal hypoglycemia based on improved understanding of molecular mechanisms of hypoglycemia was published by Stanley et al. This review makes a robust case that most forms of neonatal hypoglycemia, including transitional hypoglycemia, are due to hyperinsulinism. They show that rat newborn pancreatic beta-cell islets have lower glucose-stimulated insulin secretion than older infants and children, which is due to delayed trafficking of K_ATP_ channels from the cytosol to the cell membrane, or to genetic abnormalities in the channel itself, thereby leading to unremitting insulin secretion and hypoglycemia. Finally, they recommend screening for pathological hypoglycemia by measuring both glucose and ketone concentrations to identify infants with persistent hyperinsulinemic hypoglycemia before brain injury occurs.

This collection of manuscripts highlights some of the persisting controversies surrounding neonatal hypoglycemia, including that school age academic performance and long-term neurodevelopment is not different between transiently asymptomatic hypoglycemic newborns and non-hypoglycemic at-risk newborns (4), and treatment does not confer long-term benefits (6). To expect a single point measurement of blood glucose in a range of patients, with no assessment of the complex metabolic milieu, to be predictive of long-term outcomes is potentially naive. Instead of adding additional eminence-based recommendations on screening and management of neonatal hypoglycemia, the authors of this editorial advocate for developing consensus on priorities for the future to address these controversies.
